# Atheromatosis of the Scalp: A Novel Feature of Chronic Progressive External Ophthalmoplegia Plus Due to a Single Mitochondrial DNA Deletion

**DOI:** 10.7759/cureus.20641

**Published:** 2021-12-23

**Authors:** Josef Finsterer

**Affiliations:** 1 Neurology, Neurology & Neurophysiology Center, Vienna, AUT

**Keywords:** phenotypic heterogeneity, phenotype, psoriasis, respiratory chain, atheroma, mitochondrial, multisystem, ophthalmoplegia, deletion, mtdna

## Abstract

Chronic progressive external ophthalmoplegia (CPEO) manifests phenotypically as ptosis with ophthalmoplegia or CPEO-plus, with the affection of muscles or organs other than the extra-ocular eye muscles. Herein, a case of CPEO-plus caused by a single mitochondrial DNA (mtDNA) deletion is represented, along with several previously unreported phenotypic features. The patient is a 76-year-old Caucasian female who had experienced slowly progressive bilateral ptosis since the age of 15, followed by gradual ophthalmoparesis without double vision. Since the age of 56, she had developed mild quadriparesis, depression, easy fatigability, hypersomnia, a facial tic, optic atrophy, cataract, glaucoma, hepatomegaly, hepatic steatosis, cholecystolithiasis, diverticulosis, hyperhidrosis, mild hyper-creatine-kinase-emia, hyperlipidemia, and hyperuricemia. Moreover, she had faced previously unreported manifestations of mitochondrial disorders, psoriasis, and multiple scalp atheromas. The phenotype and a single 5kb mtDNA deletion were employed to diagnose CPEO-plus. This case demonstrates that the phenotypic spectrum of CPEO-plus is broader than expected, that psoriasis and scalp atheromas are unique features of a mitochondrial disorder, and that CPEO progresses to CPEO-plus during the years.

## Introduction

Chronic progressive external ophthalmoplegia (CPEO) expresses phenotypically as either ptosis with ophthalmoplegia or CPEO-plus, with the affection of muscles or organs other than the extra-ocular eye muscles [1]. CPEO is caused by one or more mitochondrial DNA (mtDNA) deletions [2], mtDNA point mutations [3,4], mtDNA duplications [5,6], or mtDNA depletion [7]. Mutations in nuclear DNA genes are responsible for mtDNA maintenance and function, such as POLG1, TWNK, DGUOK, RRM2B, OPA1, SUCLG1, TYMP, LIG3, FBXL4, BRCA2, and others, resulting in multiple mtDNA deletions or mtDNA depletion. In the present study, we evaluated a case of a patient with CPEO caused by a single mtDNA deletion that progressed to CPEO-plus with several previously unreported phenotypic features over the years.

## Case presentation

The patient is a 76-year-old Caucasian female with 174cm of height and 65kg of weight who had gradually developed progressive bilateral ptosis since the age of 15, followed by incremental ophthalmoparesis without double vision. She was diagnosed with struma multi-nodosa and hypothyroidism at the age of 38, which is why she started taking levothyroxine. At the age of 42, her vital capacity was reduced by half. Elevated liver function parameters, hepatomegaly, and steatosis hepatitis were identified for the first time at the age of 48. In addition, she noticed slowly progressive muscle weakness in her upper limbs since the age of 56. Moreover, she experienced hypersomnia (10-11 hours sleep per day), tics of the right face, left optic atrophy, bilateral cataract requiring surgery, glaucoma, chronic gastritis, cholecystolithiasis requiring cholecystectomy, diverticulosis, recurrent diarrhea, easy fatigability, hyperhidrosis, psoriasis, multiple atheromas of the scalp, spontaneous lumbar vertebrum 2 fracture, mildly elevated creatine-kinase (CK), hyperlipidemia, and hyperuricemia (Tab. 1). According to the clinical presentation, a mitochondrial disorder (MID) was suspected, and a work-up was initiated at the age of 49. The family history was negative for MID.

**Table 1:** Table 1. Phenotypic features of CPEO-plus in general and in the index patient BGC: basal ganglia calcification, GID: gastro-intestinal dysmotility, KSS: Kearns-Sayre syndrome

Feature	Study in which previously reported [reference number]	Index case	KSS
Ptosis	[1]	yes	yes
Ophthalmoparesis	[1]	yes	yes
Cataract	[8]	yes	yes
Optic atrophy	[9]	yes	no
Quadriparesis	[10]	yes	yes
Hypothyroidism	[11]	yes	yes
Hyperlipidemia	[12]	yes	no
Cardiomyopathy	[13]	yes	yes
Depression	[14]	yes	no
Exercise intolerance	[14]	yes	yes
GID	[14]	yes	no
Parkinsonism	[14]	no	no
Diabetes	[14]	no	yes
Cognitive impairment	[14]	no	yes
Ataxia	[14]	no	yes
Retinal degeneration	[15,16]	no	yes
Migraine	[17]	no	yes
Epilepsy	[18]	no	yes
Neuropathy	[19]	no	no
Renal failure	[14]	no	yes
Hypersomnia	no	yes	yes
Facial tic	no	yes	no
Glaucoma	no	yes	yes
BGC	no	yes	yes
Struma multinodosa	no	yes	no
Hepatomegaly	no	yes	yes
Steatosis hepatis	no	yes	yes
Diverticulosis	no	yes	no
Chronic gastritis	no	yes	no
Hyperhidrosis	no	yes	no
Atheromatosis	no	yes	no
Psoriasis	no	yes	no

**Figure 1:**
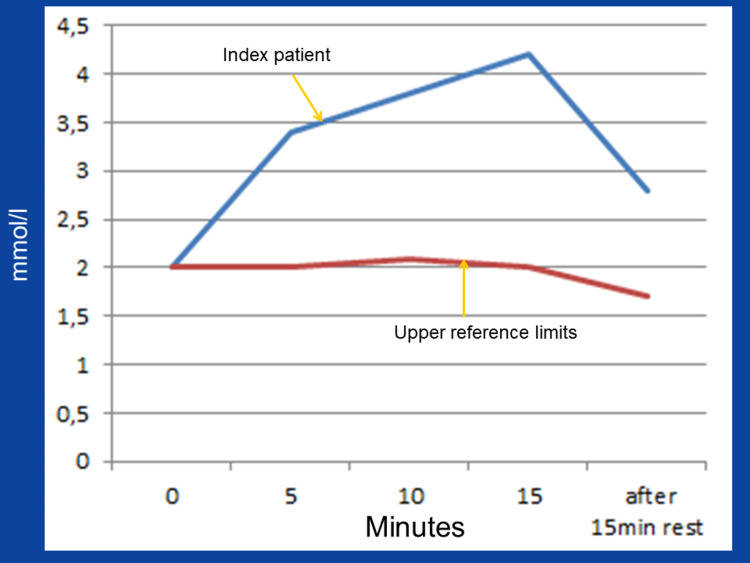
Results of the lactate stress test on a bicycle ergometer at a sub-threshold, constant workload of 30W demonstrating exercise-induced increase of serum lactate at a constant exercise below the anaerobic threshold

**Figure 2:**
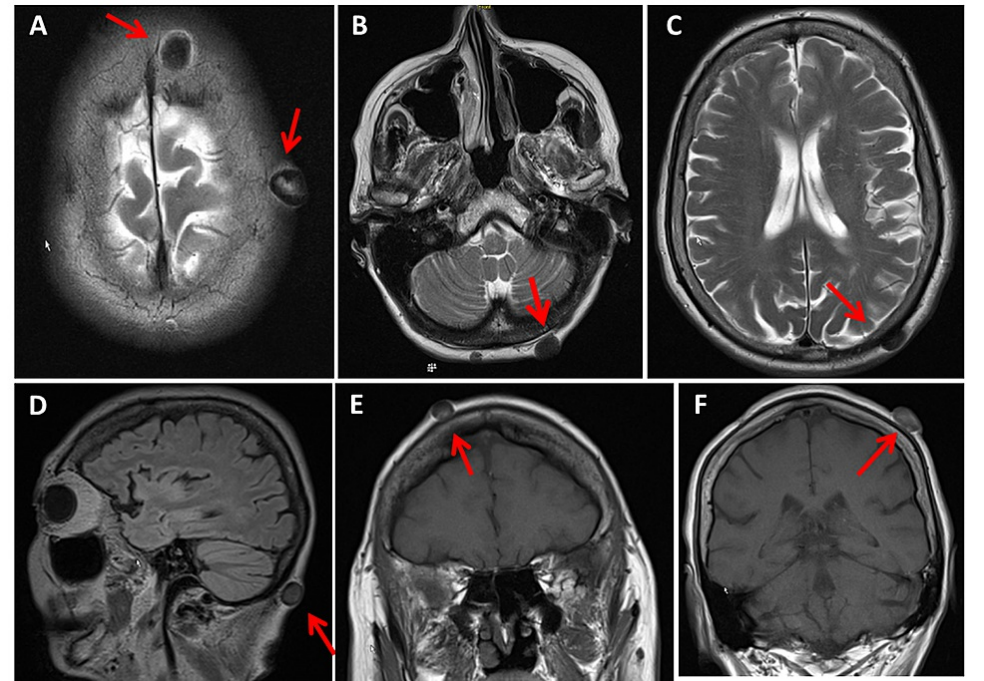
MRI of the brain showing multiple atheromas irregularly distributed over the scalp A to C: axial images showing atheromas at supra- and infratentorial levels, D: sagittal image showing a nuchal atheroma, E and F: coronary views showing atheromas lateral to the vertex

Clinical neurologic examination revealed a depressive mood, significant bilateral ptosis, ophthalmoplegia with divergent bulbs, and quadriparesis M5-. In addition, mild hyper-CK-emia (113U/l, n, <70U/l), hyperlipidemia, and vitamin-D deficiency were all present. The lactate stress test resulted in a high level of abnormality (Fig. 1). Needle electromyography from the right brachial biceps muscle was normal. Visually-evoked potentials illustrated a significant prolongation of the P100-latency to 186ms on the left side and 172ms on the right. Cerebral CT indicated mild basal ganglia calcifications as well as multiple scalp atheromas. MRI of the brain also confirmed the scalp atheromas, but the results were otherwise inconclusive (Fig. 2). An MRI of the cervical spine figured out C5/6 and C6/7 vertebrostenosis without myelopathy. Carotid ultrasound and transcranial duplex sonography were inconclusive. Echocardiography also displayed only mild left ventricular hypertrophy but normal systolic function. Furthermore, repeated 24-hour ECG showed only occasional supraventricular ectopic beats.

At the age of 49, a muscle biopsy demonstrated numerous ragged red fibers, an increased number of lipid droplets, and dysmorphic mitochondria. There were no COX-negative muscle fibers. A biopsy of the extra-ocular muscles confirmed the previous findings by the age of 59. The first genetic testing for mtDNA point mutations and mtDNA deletions in lymphocytes using a Southern blot at the age of 57 was non-informative. A second genetic examination of muscle mtDNA through a long-range polymerase chain reaction and a Southern blot revealed a single 4977bp mtDNA deletion (common deletion) with heteroplasmy rates of 60% and 30% in the skeletal and extra-ocular eye muscles, respectively. Again, the mtDNA deletion was not found in blood lymphocytes. The first-degree relatives were not tested for the causative mtDNA deletion.

She had a squint operation at the age of 59 for ophthalmoplegia. In addition, she had two ptosis correction surgeries, both of which provided only temporary relief. Her current medications were L-thyroxine, ciprasidone, tritazidone, and timolol eye drops. Anti-oxidants, cofactors, and other supplementary agents had no effect.

## Discussion

This case displays that CPEO caused by a single mtDNA deletion can have a stable course over 40 years, with ptosis and ophthalmoparesis as the only manifestations prior to the development of CPEO-plus. CPEO-plus may manifest not only with ptosis, ophthalmoparesis, ataxia, migraine, epilepsy, optic atrophy, cataract, retinal degeneration, quadruparesis, hypothyroidism, hyperlipidemia, cardiomyopathy, and neuropathy [8-19], but also with previously unreported features such as hypersomnia, facial tics, glaucoma, basal ganglia calcification, struma, hepatopathy, diverticulosis, chronic gastritis, hyperhidrosis, scalp atheromatosis, and psoriasis. Moreover, CPEO-plus can be thought of as a bridge between pure CPEO and Kearns-Sayre syndrome (KSS), as many of the CPEO-plus characteristics are also found in KSS patients.

It is unclear whether all unreported phenotypic features are genuinely attributable to the mtDNA deletion because some have not been previously reported in association with CPEO-plus and may be due to other causes and thus a coincidental finding. Meanwhile, arguments in favor of a relationship between these new features and the mtDNA deletion include the fact that at least some of these properties had previously been detected in MIDs other than CPEO and that no other plausible causes other than the mtDNA deletion were evident.

Excessive daytime sleepiness has been reported in mitochondrial depletion syndromes [20] as well as mitochondrial encephalopathy, lactic acidosis, and stroke-like episodes (MELAS) syndrome. Tic disorders have recently been discovered in Chinese patients with mitochondrial tRNA gene variants [21]. Glaucoma has been declared in a number of MIDs, both syndromic and non-syndromic. With or without hypoparathyroidism, basal ganglia calcification is another phenotypic characteristic shared by various MIDs. Endocrinopathies, including goiter and hypothyroidism, are common in MIDs. POLG1 mutations cause hepatopathy with or without steatosis in several mitochondrial depletion syndromes. Gastrointestinal compromise is prevalent in MIDs and can even be the dominant feature, as in the case of mitochondrial neuro-gastro-intestinal encephalopathy (MNGIE). TARS-2 variants have been linked to increased sweating (hyperhidrosis), which may be associated with or without dysautonomia. Despite being related to mitochondrial dysfunction, psoriasis has not been reported in either syndromic or non-syndromic MIDs. Skull atheromatosis is another feature of the index patient that has not been shown in previous CPEO-plus cases, KSS patients, or other MIDs. Scalp atheromatosis is associated with an increase in the frequency of neoplasms in MIDs. It is unknown whether the patient will experience additional reported or unreported CPEO features in the future, but given the previous disease course, the phenotype is probable to progress, possibly to KSS-like features.

Progression of CPEO to CPEO-plus has been only occasionally reported. In a study of patients with CPEO by Bromfield et al. one patient developed adrenal insufficiency at the age of 5y and a second patient developed limb muscle weakness at the age of 14y. In the study by Mancuso et al. the number of phenotypic manifestations other than CPEO increased among 228 patients with patients carrying a single mtDNA deletion over an observational period of 19y.

## Conclusions

The evaluated case demonstrates that the phenotypic spectrum of CPEO-plus is much broader than previously thought, that psoriasis and scalp atheromas are unique features of CPEO-plus, that CPEO evolves into CPEO-plus over the years, and that single mtDNA deletions may be missed in blood lymphocytes but detected in muscle cells.
